# Inequalities in the economic consequences of depression and anxiety in Europe: a systematic scoping review

**DOI:** 10.1093/eurpub/ckz127

**Published:** 2019-07-13

**Authors:** Anna Linder, Ulf-G. Gerdtham, Nadja Trygg, Sara Fritzell, Sanjib Saha

**Affiliations:** 1 Health Economics Unit, Department of Clinical Sciences (Malmö), Lund University, Lund, Sweden; 2 Department of Economics, Lund University, Lund, Sweden; 3 Epidemiology and Global Health Unit, Department of Public Health and Clinical Medicine, Umeå University, Umeå, Sweden; 4 Department of Public Health Science, Karolinska Institute, Solna, Sweden

## Abstract

**Background:**

Depression and anxiety are associated with adverse outcomes in educational achievements and economic performances. Moreover, the prevalence of these disorders is unequally distributed among different population subgroups. Our objective is to investigate whether the economic consequences of depression and anxiety differ between population subgroups of different gender, socioeconomic status (SES), ethnicity and age, in Europe.

**Methods:**

A systematic scoping literature review was performed to identify studies where exposure to depression or anxiety was identified at baseline and consequences in education, sickness absence, disability pension, unemployment and income/earnings were measured at follow-up.

**Results:**

Seventeen articles were included in this review and most of these were conducted in the Nordic countries. The consequences of depression and anxiety were stratified by gender in most of the articles. However, only in a few studies, the findings were stratified by SES, age and ethnicity. The negative consequences of depression in educational performance, disability pension and income are larger for men compared to women. Moreover, low SES individuals have more depression- and anxiety-related absence from work than high SES individuals.

**Conclusion:**

Our findings imply that the economic consequences of depression differ between population subgroups in Europe. This could have an impact on social stratification, shifting people who experience mental ill-health to lower SES groups or reinforcing an already disadvantaged position. More research is needed on unequal economic consequences of depression and anxiety in different population subgroups in Europe.

## Introduction

Overall population health indicators measured by life expectancy and healthy life expectancy are improving globally.^1^ Yet, the burden of mental disorders continues to grow and cause substantial costs to societies, for example in terms of sickness benefits and loss of productivity.^2^ Mental disorders are a leading cause of disability worldwide.^2^ In Europe, unipolar depression is the single leading cause of years lived with disability, and anxiety disorders rank as the sixth leading contributor.^3^

A large body of literature has linked mental ill-health with adverse outcomes in several aspects of life. For example, childhood and adolescent depression are associated with fewer years of schooling,^4^ which affects opportunities of human capital accumulation and future earnings.^5^^,^^6^ Depressed and anxious workers have poor labour market outcomes,^7–9^ and depression is associated with several chronic diseases which reduce the overall quality of life^10^ and increase healthcare utilization.^11^ Furthermore, depression is associated with increased mortality.^12^ Mental ill-health affects the entire population irrespective of gender, age, socioeconomic status (SES) and ethnicity. Yet, the prevalence is not equally distributed between different population subgroups or social positions, categorized by, for example education and employment status. Women and individuals from low SES are more likely to be depressed or anxious than men and individuals from high SES,^13^^,^^14^ and the prevalence of depression and anxiety is different in different ethnic groups.^15^ Moreover, for most individuals who suffer from depression and anxiety during lifetime the problems occur already in young ages (after mid-20s occurrences are mostly comorbid conditions).^16^

It is known that the prevalence of depression and anxiety is unequally distributed between population subgroups and that depression and anxiety are associated with unfavourable consequences in social and economic living conditions, health and survival. However, less is known on how the negative effects of depression and anxiety are distributed among population subgroups.

Therefore, the objective is to investigate whether educational and labour market consequences of depression and anxiety differ between men and women, and between different age groups, SESs and ethnicities in Europe.

Some reviews have previously investigated the consequences of depression and anxiety in labour market outcomes,^7–9^ and educational consequences due to mental disorders related to substance abuse.^17^^,^^18^ However, to our knowledge, no other literature review has investigated the consequences of depression and anxiety in several economic outcomes with the specific objective to study these consequences in the light of inequality. This review will inform decision-makers regarding which groups are particularly disadvantaged, and thus help in policy prioritization.

## Methods

### Theoretical framework

**Figure 1:**
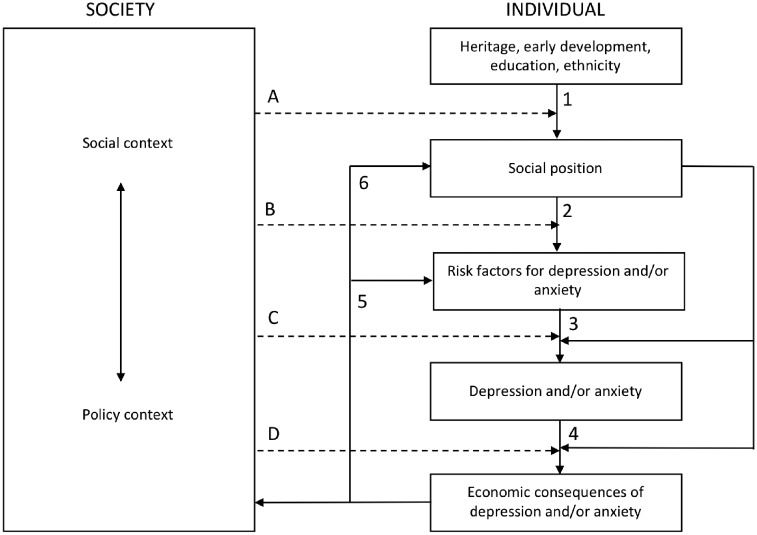
Model on health inequality determinants. *Source:* Adapted from Diderichsen et al. 2012^19^ (dashed arrows represent potential intervention channels)

We apply a model developed by Diderichsen et al.^19^ on the determinants of health inequalities to understand how social position affects individual health directly and indirectly through unequal economic consequences of mental ill-health (figure 1). Predetermined characteristics related to heritage, early development, education, and ethnicity create a range of social positions (arrow 1).^19^ Different social positions are to a varying degree exposed to the risk factors for depression and anxiety which causes a higher risks of depression and anxiety in some disadvantaged groups (arrow 2). These risk factors are, in general, socially skewed.^19^ For example, the exposures to poor physical health and precarious employment conditions (e.g. non-permanent contract) are in general higher in low SES groups. Furthermore, being exposed to several risk factors at the same time may increase the vulnerability to depression and anxiety (arrow 3). The economic consequences of these disorders may also be more severe in lower SES groups, as social position may impact on access to treatment and rehabilitation as well as labour market opportunities for individuals with reduced work capacity (arrow 4). Also, the social insurance schemes covering economic losses in times of illness may work differently for different social positions, for example if those with higher education or income have a better knowledge about rules, regulation, personal rights, etc. Unfavourable economic consequences of depression and anxiety increase the exposure to risk factors for these disorders (arrow 5), possibly worsening mental health in these groups further. Thus, if the negative economic consequences of depression and anxiety are larger among those already disadvantaged, it could cause further social stratification by shifting individuals into lower socioeconomic positions or reinforcing an already disadvantaged position (arrow 6). An impaired socioeconomic position again means a larger exposure to risk factors for depression and anxiety. This demonstrates how the bidirectional relationship between mental ill-health and adverse economic consequences causes a negative downward spiral of socioeconomic and health disadvantages, and how this spiral could be reinforced through differential exposure to risk factors between different social groups. Exposure to risk factors, the prevalence of mental ill-health and negative economic consequences are thus likely to be more common in certain disadvantaged groups, such as the low-educated or low-income earners. This theoretical framework emphasizes, however, that the spiral could be broken, and that health inequalities could be reduced by intervention on the mechanisms that cause inequalities in health.

Four potential policy intervention channels for addressing health inequalities are presented in this model.^19^ Health inequalities could be intervened by acting on social stratification (arrow A), for example by granting access to education for young people from all social backgrounds and providing support for those who need it, or by reducing the exposure and vulnerability to factors such as precarious work or financial difficulties increasing the risk of mental ill-health among some disadvantaged groups (arrows B and C). It could also be done by reducing the differential social and economic consequences of mental ill-health (arrow D), for example by providing rehabilitation or labour market opportunities for those with reduced work ability. Clearly then, from a policy perspective, it is important to know how the economic consequences of mental ill-health differ between population groups such that social and mental healthcare policies can be developed to counteract inequalities in society. This can be done not only by addressing inequalities in risk factors for these disorders but also by addressing the inequalities in the consequences of these disorders.

### Search

The review was conducted according to the methodological guidelines for literature reviews produced by the Public Health Agency of Sweden^20^ and reported according to the Preferred Reporting Items for Systematic Reviews and Meta-analyses: the PRISMA statement.^21^ The search process, study selection and quality assessment of the individual studies were systematic in their structures, while the synthesis of the results followed a more narrative approach. This means we did not use any statistical method (e.g. meta-analysis) to summarize the results, which would have been difficult with the broad scope of our research question. No review protocol was registered.

A systematic search with keywords was performed in the electronic databases MEDLINE (PubMed), Web of Science, EconLit, Cumulative Index to Nursing and Allied Health (CINAHL) and PsycINFO. In addition to the electronic databases, we searched the home pages of the World Bank, the World Health Organization (WHO), the UK Department for International Development (DFID) and the Organization for Economic Co-operation and Development (OECD). Moreover, we performed backwards and forward snowball searches in reference lists and citation records of the included studies. A detailed search strategy including the full search strings is provided in the Supplementary Materials. We searched for articles published in Swedish or English between January 2007 and December 2016.

### Study selection

**Figure 2:**
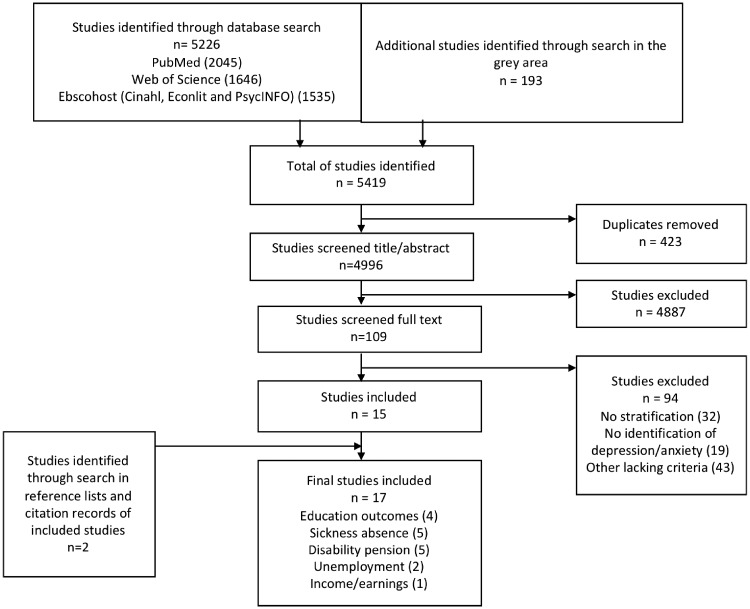
Flow diagram of identification, screening and inclusion/exclusion of studies

Initial total hits from each database were exported into EndNote and duplicates were removed. Selection of relevant studies was based on the following PECOS inclusion and exclusion criteria (presented in detail in table 1): (i) the study was performed in a European Union country (EU28), Norway, Switzerland or Iceland; (ii) exposure to depression or anxiety was measured at baseline; (iii) educational performance and labour market outcomes (in sickness absence, disability pension, unemployment or income/earnings) were measured at follow-up; (iv) the results were presented in considered strata by gender, age, SES or ethnicity; and (v) the study was of longitudinal or case–control design. The study selection was performed by the two co-authors (A.L. and S.S.) separately and uncertainty was resolved by discussion. The study selection process was performed in two phases, first through title- and abstract-screening, and later by full-text reading. A detailed flowchart of the study selection procedure is presented in figure 2.

**Table 1:** PECOS inclusion/exclusion criteria

Criteria		Inclusion	Exclusion
Population	Title/abstract	European population^a^	Non-European population
	Full text	Population stratified by any subgroup of gender, SES, age or ethnicity	European population^a^ not stratified by gender, SES age or ethnicity
Exposure	Title/abstract	Mental illness, depression and/or anxiety	Other health problems
	Full text	Depression and/or anxiety, symptoms identified with validated symptom scale instrument, or diagnosis identified with diagnostic system-based interview, or sickness absence certified	Depression and/or anxiety identified as a comorbidity with other mental disorder
Comparator	Title/abstract	European population^a^	Non-European population
	Full text	Comparing population subgroup of gender, SES, age or ethnicity	European population^a^ not stratified by gender, SES age or ethnicity
Outcomes	Title/abstract	Educational performance; sickness absence; disability pension; unemployment; income/earnings	Other than listed under inclusion criteria
	Full text	Same as above	Same as above
Study design	Title/abstract	Observational studies	Intervention studies
	Full text	Longitudinal studies; case–control studies	Cross-sectional studies

^a^European Union countries (EU28) and Norway, Switzerland and Iceland. European population refers to any population living in Europe.

### Data extraction

Data were extracted from the selected articles by the two co-authors (A.L. and S.S.). Information was extracted on several issues such as first author, year of publication and country where the study was conducted. Type of exposure, the instrument used to identify depression and/or anxiety, study population, sample size, study design, duration of the study and definition of outcome measures were moreover extracted. Key findings on the economic consequences of depression and/or anxiety were extracted for each subgroup separately. Estimate sizes were extracted as these were reported in the selected articles, for example in odds ratios, hazard ratios or the beta coefficients (β), as well as the 95% confidence intervals (CI) or *P*-values. If several statistical models were presented in an article, the results from the preferred model (by the authors) were reported. If no preference was stated by the authors, the estimates from the most comprehensive model were extracted.

### Quality assessment

The quality assessment was based on guidelines by the Effective Public Health Practice Project (EPHPP) group.^22^ According to the EPHPP, quality is rated by eight components: risk of selection bias, study design, risk of confounders, blinding, data collection methods, drop-outs/withdrawals, intervention integrity and analysis. Since the original outline of the EPHPP guideline is more suitable for intervention trials, it was adjusted to fit the purpose of this review. Blinding and intervention integrity components were excluded. The component which rated drop-out/withdrawals was interpreted to assess attrition of the sample at follow-up. Each component of quality was rated as strong (1), moderate (2) or weak (3) based on the evidence. For example, the component for attrition was rated by the percentage of participants at follow-up [>80% (1), 60–79% (2), <60% (3)] and if the reasons for any attrition were provided. An overall rating for risk of bias was given based on the component ratings; studies with zero/one/≥two weak components were rated with low/moderate/high risk of bias (see table 2).

**Table 2:** Detailed characteristics and key findings of the included studies

First author; Year of publication; Country	Exposure; Instrument of identification	Study population; Sample size; Number of cases	Study design; Duration to follow-up; Attrition at follow-up	Definition of outcome	Results	Risk of bias^a^
Jonsson et al.^26^; 2010; Sweden	Depression; BDI and CES-DC	First year students in upper secondary school (population-based); *n* = 609; Cases = 361	Longitudinal prospective study; 15 years; 3.4%	Completed degree from university or college at age 30	Depression was associated with decreased educational performance only in boys, OR 0.27, 95% CI 0.08–0.93	Low; 1, 2, 1, 1, 1, 1
Verboom et al.^23^; 2014; The Netherlands	Depressive problems; YSR and CBCL	Children and adolescents in the TRAILS study (population-based); *n* = 2230; Cases = 24%	Longitudinal prospective cohort; 1st follow-up 2 years 2nd follow-up 4 years; 30% (2 years) 58% (4 years)	Teacher reports	Depression was associated with decreased educational performance only in girls, β = −0.065, *P* < 0.05 (1st follow-up), β = −0.082, *P* < 0.05 (2nd follow-up)	High; 2, 2, 3, 2, 3, 1
Rothon et al.^24^; 2009; UK	Depressive symptoms; SMFQ	Adolescents in the RELACHS study (representative); *n* = 1636; Cases = 465	Longitudinal prospective cohort; 2 years; NM	GCSE results	Depression was significantly associated with decreased educational performance only in boys, OR 0.58, 95% CI 0.43–0.79 The impact of depression on educational performance did not differ significantly by SES The impact of depression on educational performance differed significantly with ethnicity, OR (Bangladeshi girls) 0.29, 95% CI 0.17–0.52	High; 2, 2, 3, 1, 3, 1
Riglin et al.^25^; 2013; UK	Depressive symptoms; SMFQ General anxiety; SCARED	Students in secondary school (type not clear); *n* = 262; NM	Longitudinal; <1 year; 22%	Attainment in school	Depression was significantly associated with decreased educational performance only in boys, β = −0.21, *P* < 0.05 Anxiety was not significantly associated with educational performance in boys or girls	Moderate; 2, 2, 3, 1, 2, 2
Koopmans et al.^29^; 2010; The Netherlands	Depressive and anxiety disorders; Diagnosis according to ICD-10	Employees in Dutch post and telecommunication companies (population-based); *n* = 137 172; Cases = 1025 (depressive episodes) 429 (anxiety episodes)	Longitudinal; 7 years; No loss	Onset, duration and recurrence of sickness absence (medically certified, usually >14 days absence)	Depression and anxiety was significantly associated with increased onset (ID)^b^ and recurrence (RD)^b^ of SA in both genders. RD was similar between genders and ID was higher in women RD (women) 78.9, 95% CI 64.1–93.7 (men) 83.8, 95% CI 71.9–95.7, ID (women) 5.2, 95% CI 4.7–5.6 (men) 3.0, 95% CI 2.8–3.2 The association between depression and anxiety and duration of SA was similar between men and women. The association between depression and anxiety and onset of SA increase with age among men ID (age <35) 1.6, 95% CI 1.3–1.9 (age 35–44) 3.6, 95% CI 3.1–4.1 (age 45–54) 3.7, 95% CI 3.3–4.1 (age >54) 3.0, 95% CI 2.4–3.6. The association between depression and anxiety and onset and recurrence of SA decreased with age among women ID (age <35) 5.1, 95% CI 4.4–5.9 (age 35–44) 5.8, 95% CI 5.0–6.5 (age 45–54) 4.9, 95% CI 4.1–5.7 (age >54) 3.2, 95% CI 2.0–4.4, RD (age <35) 90.4, 95% CI 30.2–150.6 (age 35–44) 90.3, 95% CI 52.1–128.5 (age 45–54) 53.5, 95% CI 10.9–96.1 (age >54) 66.1, 95% CI 0–417.2	Moderate; 2, 2, 3, 1, NA, 1
Knudsen et al.^30^; 2013; Norway	Depressive and anxiety symptoms; HADS	Employees in the HUSK study (population-based); *n* = 13 436; Cases = 1485 (anxiety) 452 (depression) 652 (CMDA)	Longitudinal prospective cohort; 6.2 years; No loss	Onset of sickness absence >15 days	CMDA was associated with increased onset of SA in both genders, HR (men) 1.41, 95% CI 1.21–1.65 (women) 1.25, 95% CI 1.08–1.44 Anxiety was associated with onset of SA only in women, HR 1.20, 95% CI 1.09–1.31 Depression was not significantly associated with SA in men or women Gender interaction effects were insignificant in all specifications (*P* > 0.9)	Low; 2, 2, 1, 1, NA, 1
Lexis et al.^28^; 2009; The Netherlands	Depressive complaints; HADS	Employees in the Maastricht Cohort Study (type not clear); *n* = 3339; Cases = 619	Longitudinal prospective cohort; 10 months; No loss	Onset of sickness absence (total number of days absent over 10 months)	Depression was significantly associated with increased onset of SA in both genders. The association was similar between men and women, β (men) = 0.0735, 95% CI 0.0549–0.0921; β (women) = 0.0730, 95% CI 0.0366–0.1095	Low; 1, 2, 1, 1, NA, 1
Virtanen et al.^27^; 2011; Finland	Depressive and anxiety disorders; Diagnosis according to ICD-10	The Finnish Public Sector Study Cohort (1997–2005) (population-based); *n* = 141 917; Cases = 2679 (depression) 314 (anxiety)	Longitudinal prospective cohort; 9 years; No loss	Onset and duration of long-term work disability^c^ >90 days	Low SES^d^ increased onset of work disability^c^ following depression, HR 1.23, 95% CI 1.10–1.37 and anxiety, HR 1.41, 95% CI 1.04–1.93 High SES^d^ increased return to work (decreased duration) of work disability^c^ following depression, HR 1.45, 95% CI 1.26–1.67 SES did not significantly affect the association between anxiety and duration of work disability^c^	Low; 1, 2, 1, 1, NA, 1
Ervasti et al.^31^; 2013; Finland	Depressive disorder; Diagnosis according to ICD-10	The Finnish Public Sector Study Cohort (2005–2011) (population-based); *n* = 125 355; Cases = 4266	Longitudinal prospective cohort; 7 years; No loss	Onset, duration and recurrence of work disability^c^ >9 days	Low SES^d^^,^^e^ increased onset of work disability^c^ following depression RR (manual worker) 1.35, 95% CI 1.13–1.61, RR (low education) 1.52, 95% CI 1.25–1.85. Low SES^e^ increased duration of work disability^c^ following depression, RR 1.21, 95% CI 1.05–1.39. Low SES^d^ increased recurrence of work disability^c^ following depression, HR 1.14, 95% CI 1.02–1.26	Low; 1, 2, 1, 1, NA, 1
Wedegaertner et al.^36^; 2013; Germany	Depressive and anxiety disorders; In- and outpatient diagnosis according to ICD-9	Employees with statutory health insurance (type not clear); *n* = 125 019; Cases = 1500	Longitudinal prospective cohort; 6.4 years; No loss	Disability pension	Depression and CMDA were associated with increased DP in both genders. The associations were higher in men, for inpatient depression HR (men) 3.78, 95% CI 2.85–5.01, (women) 2.46, 95% CI 1.70–3.56, for outpatient depression HR (men) 1.70, 95% CI 1.41–2.04, (women) 1.31, 95% CI 1.09–1.58, for inpatient CMDA HR (men) 4.13, 95% CI 3.01–5.67, (women) 2.96, 95% CI 2.08–4.20, for outpatient CMDA HR (men) 2.59, 95% CI 1.97–3.41, (women) 1.42, 95% CI 1.04–1.93. Gender interaction effect significant for outpatient CMDAAnxiety was significantly associated with DP only in women, outpatient anxiety HR 1.30, 95% CI 1.06–1.59. Gender interaction effect non-significant	Moderate; 2, 2, 3, 1, NA, 1
Lassemo et al.^33^; 2016; Norway	Depression; CIDI	Population cohort from the OsLof study (representative); *n* = 1230; Cases = 204	Longitudinal prospective cohort 10 years; 36%	Disability pension	Depression was associated with increased DP in both genders. The association was higher in men, HR (men) 2.7, 95% CI 1.2–5.7 (women) 1.6, 95% CI 1.0–2.5	Low; 2, 2, 1, 1, 2, 1
Dorner et al.^35^; 2015; Sweden	Depressive episode; Diagnosis according to ICD-10	Population aged 16–64 years (population-based); *n* = 4 823 069; Cases = 23 722	Longitudinal prospective cohort; 5 years; No loss	Disability pension	Depression was associated with increased DP in both genders. The association was higher in HR (men) 14.21, 95% CI 13.39–15.07 (women) 11.65, 95% CI 11.13–12.20	Low; 1, 2, 1, 1, NA, 1
Rytsala et al.^32^; 2007; Finland	MDD; DSM-IV by SCAN 2.0	Employees in the VDS (clinical-based); *n* = 269 (all are cases)	Longitudinal; 18 months; 21.5%	Disability pension	Male gender was associated with DP in depressed individuals, OR 0.17, 95% CI 0.035–0.84 Vocational education was not significantly associated with DP in depressed individuals Age was significantly associated with DP in depressed individuals, OR (higher age^f^) 1.15, 95% CI 1.05–1.26	Moderate; 2, 2, 3, 2, 2, 2
Holma et al.^34^; 2012; Finland	MDD; DSM-IV by SCAN 2.0	Employees in the VDS (clinical-based); *n* = 269 (all are cases)	Longitudinal; 5 years; 32.3%	Disability pension	Gender was not significantly associated with DP in depressed individuals Vocational education was associated with DP in depressed individuals OR (lack of vocational education) 2.38, 95% CI 1.08–5.2 Age was associated with DP in depressed individuals OR (age >50) 6.25, 95% CI 2.71–14.3	Moderate; 2, 2, 3, 2, 2, 2
Andreeva et al.^38^; 2015; Sweden	MDD; SCL-CD	Employees in the SLOSH (representative); *n* = 3503; Cases = 48	Longitudinal prospective cohort; 2 years; 23.3%	Unemployment in the context of downsizing	Depression was associated with increased unemployment only in women, RRR 2.18, 95% CI 1.01–4.69	Low; 2, 2, 1, 1, 2, 1
Jefferis et al.;^37^ 2011; UK, Spain, Slovenia and Portugal	MDD; CIDI	GP attendees aged 18–75 (type not clear); *n* = 1349; Cases = 218	Longitudinal prospective cohort; 2 years; 34%	Unemployment	Depression was not significantly associated with unemployment in men or women	Low; 2, 2, 1, 1, 2, 1
Hakulinen et al.^39^; 2016; Finland	Depressive symptoms; BDI	Participants in the YFS (representative); *n* = 1709; Cases = 2.21% (women); 2.03% (men)	Longitudinal prospective cohort; 28 years; NM	Earnings^g^/taxable income^h^	Depression was associated with decreased income and earnings only in men, for income β = −0.27, *P* < 0.01, for earnings β = −0.34, *P* < 0.01	Moderate; 2, 2, 1, 1, 3, 1

BDI: Beck Depression Inventory; CBCL: Child Behaviour Checklist; CES-DC: Centre for Epidemiological studies-Depression Scale for Children; CIDI: Composite International Diagnostic Interview; CMDA: comorbid depression and anxiety; DSM-IV: Diagnostic and Statistical Manual for Mental Disorders; DP: disability pension; GCSE: General Certificate of Secondary Education; GP: General Practice; HADS: Hospital Anxiety and Depression Scale; HR: hazard ratio; HUSK: Hordaland Health Study; ICD-10: International Statistical Classification of Diseases and Related Health Problems-Tenth Revision; ICD-9: International Statistical Classification of Diseases and Related Health Problems-Ninth Revision; ID: incidence density; MDD: major depressive disorder; NA: not applicable; OR: odds ratio; RD: recurrence density; RELACHS: Research with East London Adolescents: Community Health Survey; RR: rate ratio; RRR: relative risk ratio; SA: sickness absence; SCAN 2.0: WHO Schedule for Clinical Assessment in Neuropsychiatry; SCARED: Screen for Child Anxiety-Related Emotional Disorders; SCL-CD: Symptom Checklist-Core Depression; SLOSH: Swedish Longitudinal Occupational Survey of Health; SMFQ: Short Moods and Feelings Questionnaire; TRAILS: Tracking Adolescents Individual Lives Survey; VDS: Vantaa Depression Study; YFS: Young Finns Study; YSR: Youth Self Report.

^a^Risk of bias was assessed based on six components of quality: risk of selection bias, study design, risk of confounders, data collection methods, attrition and analysis. Each component of quality was rated as strong (1), moderate (2) or weak (3) based on the evidence, and an overall rating for risk of bias was given based on the component ratings.

^b^ID and RD per 1000 person-years.

^c^Work disability was defined as being on SA or having DP.

^d^SES was derived from occupational title classification; upper-grade non-manual, lower-grade non-manual and manual workers.

^e^SES was derived from educational level: basic, intermediate and high.

^f^The variable age was not further explained in the study.

^g^Mean annual wage and salary earnings (1993–2010).

^h^Mean annual wage and salary earnings, self-employed income, capital income, income transfers and social security benefits (1993–2010).

## Results

Seventeen longitudinal studies were included in this review. Detailed characteristics and key findings of the studies are presented in table 2.

### Consequences in educational achievements

The relationship between mental ill-health and education was studied in four articles.^23–26^ In three of these articles, the consequences of depression were studied,^23^^,^^24^^,^^26^ and in one article consequences of both depression and anxiety were studied.^25^ Gender-stratified analyses were performed in all the studies, while the impacts of SES and ethnicity were investigated in only one study.^24^ The results in three studies show that depression is associated with poorer educational performance among boys only.^24–26^ Depression was found to be associated with decreased attainment in secondary school,^25^ lower probability of completing higher education at age 30 years^26^ and lower probability of performing well on test results (General Certificate of Secondary Education, GCSE) at age 16,^24^ among boys only. In contrast, depression was found to be associated with poorer school performance among girls only when performance was measured with teacher reports.^23^ No significant association was found between anxiety and educational attainment in either boys or girls.^25^ Consequences of depression on GCSE results did not vary significantly between SESs (proxied by eligibility for free school meals),^24^ but there was some evidence that the consequences of depression varied with ethnicity. The probability of performing well on GCSE results was significantly lower following depression only among Bangladeshi girls (living in the UK), while this association was non-significant among girls of other ethnicities (ethnicities categorized by white UK, Bangladeshi, Pakistani, Asian Indian, black African, black Caribbean and black British).^24^ No significant differences based on ethnicity were found in boys.^24^

### Consequences in employment

#### Sickness absence

Sickness absence refers to a temporary absence from work due to sickness. The relationship between mental ill-health and subsequent sickness absence was examined in five studies.^27–31^ In two of these studies the consequence of depression in sickness absence was examined,^28^^,^^31^ and in three studies the consequences of both depression and anxiety were examined.^27^^,^^29^^,^^30^ Sickness absence was measured and defined in a variety of ways in the selected articles. Onset of sickness absence reflects at least one period being absent from work, but several studies used cumulative incidence during a time period to identify onset.^28^^,^^29^^,^^31^ Duration of sickness absence reflects the length of the absent period until return to work, and recurrence of sickness absence reflects having a new incidence within a time period of a first episode. Consequences in work disability were measured in two studies, which were defined as being on sickness absence or having disability pension.^27^^,^^31^ Gender-stratified analyses were performed in three studies.^28–30^ The results of these studies suggest that the risks of sickness absence following depression and comorbid depression and anxiety (CMDA) are similar between genders.^28–30^ In one study, anxiety was associated with higher sickness absence only among women, but gender interaction effects were non-significant implying no differences between men and women.^30^ A slightly higher risk of onset of sickness absence was found in women compared to men, but it is not stated if the difference between gender is statistically significant. However, when depression and anxiety are measured in combination with stress-related disorders women have a significantly higher onset of sickness absence than men.^29^ In the same study, it was found that onset and recurrence of sickness absence decrease with age among women, and that onset of sickness absence increase with age among men.^29^

Socioeconomic inequalities in the risk of work disability were examined in two studies; Ervasti et al.^31^ studied depression-related work disability >9 days, while Virtanen et al.^27^ studied long-term anxiety- or depression-related work disability >90 days. The results suggest that depression- and anxiety-related work disabilities are influenced by a socioeconomic gradient. Low SES was proxied by both occupation type and education level. Manual workers have increased risks of onset and recurrence of work disability, and a decreased chance of return-to-work, compared to non-manual workers (higher and lower grade non-manual work).^27^^,^^31^ It is possible that these consequences also depend on type of job within the different work categories but this was not further explored. The lower educated individuals have a higher risk of onset of work disability and longer durations to return-to-work compared to higher educated individuals.^31^ The educational gradient was stronger compared to the occupational gradient for onset of work disability.^27^^,^^31^

#### Disability pension

Consequences of depression and anxiety on disability pension were studied in five articles.^32–36^ Disability pension refers to being granted pension due to permanently impaired work capacity by injury or disease. In four of these articles, the consequence of depression was studied,^32–35^ and in one article the consequences of depression, anxiety and CMDA were studied.^36^ Gender-stratified analyses show that impacts of depression and/or CMDA on disability pension were higher among men.^33^^,^^35^^,^^36^ An increased risk of disability pension among women with anxiety was noted, but the impact was small in relation to the consequences of depression and CMDA, and gender interaction effects were non-significant. However, in a Finnish sample of depressed individuals, male gender was associated with lower disability pension rates at 18 months follow-up^32^ while gender was non-significant at five years follow-up.^34^ Moreover, the risk of being on disability pension increased significantly with age^32^^,^^34^ and with lack of vocational education.^34^

#### Unemployment and income/earnings

Consequences of depression on unemployment were examined in two studies.^37^^,^^38^ In one study, depression was significantly associated with higher unemployment in women only.^38^ In the other study, no significant association between depression and unemployment was found in men or women after controlling for education level and baseline employment status.^37^ Consequences of depression in income and earnings were examined in one study, depression was found to be associated with decreased earnings and lower income in men but not in women.^39^

### Quality of included studies

Risk of bias was high in two studies,^23^^,^^24^ moderate in six studies^24^^,^^25^^,^^29^^,^^32^^,^^34^^,^^36^^,^^39^ and low in the remaining nine studies.^26–28^^,^^30^^,^^31^^,^^33^^,^^35^^,^^37^^,^^38^ Risk of bias was mostly related to the lack of controlling for potential confounders, for example prior educational performance^23^ and physical health,^32^^,^^34^^,^^36^ and attrition.^23^^,^^24^^,^^39^ Attrition ranged from zero in those studies that linked to registers and insurance records to 58% in one study where more than half of the sample was lost at follow-up.^23^ In two studies attrition was selective^24^^,^^39^ such that those with mental ill-health were more likely to be missing at follow-up compared to the healthy control group. Poor statistical power related to small sample sizes was also an issue.^32^^,^^34^

## Discussion

We performed a systematic scoping literature review of longitudinal studies where exposure to depression and/or anxiety was identified at baseline and economic consequences in education and labour market outcomes were measured at follow-up. Our aim was to investigate whether the economic consequences of depression and anxiety differ between men and women, and between different age groups, SESs and ethnicities in Europe. We chose to investigate economic consequences in education, sickness absence, disability pension, unemployment and income, since consequences in these areas are considerable and may be longstanding, both for the individual and for the society.

### Gender differences

Our findings suggest that boys suffer worse consequences than girls in terms of educational performance following depression.^24–26^ In one study the opposite was found, but in this study educational performance was reported by the class-teacher or the mentor.^23^ These reports are likely affected by teacher subjectivity and therefore less objective compared to the indicators for educational performance used in the other studies (GCSE results,^24^ attainment in secondary school^25^ and completed college degree^26^). Thus, we find it likely that boys are disadvantaged compared to girls in the relationship between depression and educational performance.

Our findings also suggest that men are more likely to hold disability pension following depression and CMDA compared to women.^33^^,^^35^^,^^36^ In one study the opposite was observed but it was not revealed if the difference in outcome (disability pension) between men and women was related to unequal consequences of depression, or only to differences in the distribution of disability pension.^32^ If the need for disability pension is similar among depressed men and women, the finding that men more often hold disability pension could reflect a disadvantage for either men or women. If women are not being granted disability pension when they are in need for it, it could reflect a poorer access to social protection among women. However, if women to a larger extent are able to return to the labour market, thus avoiding the negative economic consequences of disability pension, for example following successful rehabilitation, the situation is the opposite and this could be seen as a disadvantage among men.

One study found that depression is significantly associated with lower income and earnings only among men.^39^ Their findings are in line with a US study,^40^ however, it is difficult to make a conclusion based on just one study. There was a lack of consensus also on the association between depression and unemployment. In one study, no significant association was found^37^ while in another study, it was found that depression resulted in significantly increased risk of job loss only among women.^38^ Both studies estimated the impact of major depression but the studies were conducted in different settings, one studied general practitioner-attendees (patients) in a multi-national setting in Slovenia, UK, Spain and Portugal,^37^ while the other study among employees in the context of organizational downsizing in Sweden.^38^ The different results between these studies may reflect differences in baseline characteristics between the study populations and settings, for example regarding gender roles and access to social security, which also complicates generalization across the studies. According to Andreeva et al.,^38^ employment rates in Sweden are similar between genders, but women are more often found in part-time or temporary employment, which could explain a stronger relationship between depression and unemployment in the Swedish women. More research on the consequences of depression on employment status and income/earnings in different population subgroups in Europe is needed.

There was considerable heterogeneity between all the studies looking at gender differences in the consequences of depression and anxiety. For example, follow-up in educational outcomes varied from less than one year^25^ up to 15 years.^26^ Consequences in disability pension were considered in one study sample of 269 employees in Finland,^34^ whereas in another study, the entire Swedish population^35^ was considered. Depression and anxiety were identified in different ways; with varying instruments/diagnostic, in some studies separately and in some studies in combination (CMDA) (table 2). Moreover, the economic consequences of mental ill-health were measured with large variations, for example with different indicators for educational performance and different time perspectives for disability pension. Although a variety of measures were used to study sickness absence, it seems that the consequences in sickness absence were comparable between men and women.

Considering the level of heterogeneity between the studies and also some quality deficiencies, overall, the results suggest that boys and men suffer worse consequences following depression in several economic outcomes. There are several potential explanations for this related to health-seeking behaviour, how mental illness manifests itself, and how the social security system is constructed. These aspects differ not only between social positions (apart from the manifestation of illness) (see Diderichsen et al.^19^) but also between men and women. First, men have a lower propensity to seek or receive treatment for depression than women.^26^^,^^35^ Consequently, depression could, when detected, result in more severe consequences among men than among women.^26^^,^^35^ Second, it has been suggested that depression among men is related to alcohol problems, aggressiveness, antisocial behaviour and lowered stress tolerance more often than among women.^26^ These behaviours are likely to have an impact on education and labour market outcomes thus explaining why boys and men suffer worse consequences following depression in several economic outcomes. Third, with a higher employment rate, men are generally more likely to have better access to the social insurance schemes covering economic losses in times of illness (such as disability pension), as this is often linked to employment. Thus, the unequal economic consequences (for some outcomes) of depression between men and women seem to counteract the social stratification of economic resources between men and women and hypothetically also inequalities in mental health.

### Socioeconomic, ethnic and age-related differences

In one study it was suggested that SES and ethnicity have little impact on the relationship between depression and educational performance.^24^ It is possible that missing data, which was selective with respect to SES, had an impact on these results.^24^ Furthermore, using eligibility for free school meals as a proxy of SES among children could be questioned, since this measure possibly capture only a fraction of disadvantaged children.^24^ It was found that the negative impact of depression on educational performance was significantly higher only among Bangladeshi girls (living in the UK), compared to girls of other ethnicities. However, the ethnic subgroup composition in this study sample was not representative of national samples, for example the white British sample used in this study was more deprived than the general white British population, which may have influenced the results.^24^ Thus, the (lack of) impact of socioeconomic and ethnic components in the relationship between depression and educational performance needs to be further assessed.

As earlier mentioned, inequalities in consequences of ill mental health, according to the Diderichsen model,^19^ relates to how social position determines variations in the exposure of risk factors and vulnerability to the health effects of these, ultimately impacting on mental health and further producing differential economic consequences in different social groups. Our findings support that there are more severe consequences in lower social economic groups, and suggest that there is a socioeconomic gradient in the relationship between depression and anxiety and work disability (sickness absence and disability pension combined).^27^^,^^31^ Furthermore, the gradient was stronger when SES was proxied by education level compared to when it was proxied by occupational status, which could indicate that those lowest educated are particularly disadvantaged in the relationship between depression and work disability.^27^^,^^31^

As suggested by Diderichsen et al.,^19^ social position may impact on access to treatment and rehabilitation as well as labour market opportunities for individuals with reduced work capacity.

Poorer mental health in lower SES groups has been found in several studies.^13^^,^^14^ In line with Diderichsen et al, Virtanen et al.^27^ suggest that reasons for this include poorer physical health among low SES groups. Furthermore, that the exposure to a combination of risk factors such as fewer resources to invest in treatment, poorer adherence to treatment, less social support and greater insecurities in employment, contributes to more severe consequences of depression and anxiety among low SES groups.^27^ The finding of this review, that they also suffer worse consequences of depression in terms of work disability supports the mechanism of reinforced social stratification to the disadvantage of individuals with lower SES, in contrast to what we find on gender differences.

Finally, in one study it was suggested that the risk of sickness absence following depression and anxiety decreased with age among women but increased (for onset) among men.^29^ Thus, it seems as though gender is important to consider when studying the age differences in the risk of sickness absence following depression and anxiety, but more research is needed on these mechanisms.

### Generalizability

Criteria for inclusion in this review were European Union countries and Norway, Switzerland and Iceland. Yet, 10 out of the 17 studies were conducted in Nordic countries, and 6 out of the remaining 7 studies were exclusively performed in Western European countries. The Nordic countries are in general characterized by universal access to healthcare services and other social securities, e.g. sickness and unemployment transfers, which could have impact on the consequences of depression and anxiety.^19^ Thus, the results of this review are likely to be generalizable to settings with similar characteristics.

One study included populations from UK, Spain, Portugal and Slovenia but there seems to be a lack in the existing research focusing on these question particularly in Southern and Eastern European countries.

#### Quality assessment

Estimating the relationships between mental ill-health and educational and employment consequences is complicated by endogeneity issues. Selection bias and reversed causality may cause endogenous variation in the outcomes between the mentally ill and the healthy population. A number of the selected studies failed to address these issues, however, in general, there was only low or moderate risk of bias in the included studies (two studies had high risks of bias). To account for these issues future research will benefit from extended control of confounders, for example regarding physical health and pre-depressive conditions of the relevant outcomes.

### Strengths and weaknesses

In this review, we investigated whether the existing literature suggests that educational and employment consequences of depression and anxiety differ between population groups. On one hand, the reader is presented with a comprehensive overview of consequences in education, sickness absence, disability pension, unemployment and earnings, which could be seen as a strength of this review. On the other hand, methodological differences between the included studies, for example regarding heterogeneity in selecting the sample, sample size and validity of the different instruments used to identify depression and anxiety, prevent us to disentangle all aspects which may be of importance for the estimated relationships. Thus, our broad approach could also be seen as a weakness. Nonetheless, the fact that significant differences in the consequences of depression and anxiety exist between some of these population subgroups is an important finding in itself.

The PRISMA guidelines, as well as the guidelines for literature reviews provided by the Public Health Agency of Sweden,^20^ were followed in a systematic way, such that the search and study selection can be reproduced. Moreover, the quality assessment process was thorough and transparent, which can be seen as strengths of this review. However, it is possible that the adjustment of the quality assessment tool, as well as the quality assessment of the individual studies, is influenced by the subjectivity of the authors, which is a potential weakness. This review was originally performed for the Public Health Agency of Sweden within a Government assignment regarding determinants for inequalities in mental health. The geographical restriction was set to resemble the Swedish setting as close as possible. The geopolitical differences between the countries may have affected the results but was not further explored. The chosen time frame was set to ensure that the results reflects the current mental health situations in Europe.

## Conclusion

The negative consequences of depression in terms of educational performance and disability pension are larger for men compared to women, and there are some indications that depressed men suffer larger income losses than depressed women. Moreover, a socioeconomic gradient affects the relationship between mental ill-health and work disability such that low SES increases depression- and anxiety-related absence from work. This could have an impact on social stratification, shifting people who experience mental ill-health to lower SES groups or reinforcing an already disadvantaged position. Unequal economic consequences of depression and anxiety could thus both increase or decrease inequalities in mental health. Research is lacking on inequalities in the economic consequences of depression and anxiety in Southern and Eastern Europe and research is moreover scarce on ethnic and age-related variation in these consequences also in the rest of Europe.

## Funding

Funding was provided by the Public Health Agency of Sweden. The Health Economics Unit at Lund University also receives core funding from Government Grant for Clinical Research (ALF; Dnr F: 2014/354), and Region Skåne (Gerdtham). The funding agencies had no role in the identification, design, conduct and reporting of the analysis.

*Conflicts of interest*: None declared.

**Key points**

- Boys and men seem to suffer more severe consequences of depression in education and disability pension compared to girls and women
- Low socioeconomic status (SES) groups have more depression- and anxiety-related absence from work. Since low SES groups also have poorer mental health (in general), the unequal economic consequences of depression could reinforce the spiral of adverse relationships between mental ill-health and SES.
- Research is particularly lacking on inequalities in the economic consequences of depression and anxiety in Southern and Eastern Europe and research focussing on ethnic and age-related variations of these consequences is scarce also in the rest of Europe.

## Supplementary Material

ckz127_Supplementary_Data
