# Supplementary material for: Inequalities in the economic consequences of depression and anxiety in Europe: a systematic scoping review
Source: Eur J Public Health. 2019 Jul 13;30(4):767–77. doi: 10.1093/eurpub/ckz127 (PMC7445046; doi:10.1093/eurpub/ckz127)
Supplement: ckz127_Supplementary_Data [file ckz127_supplementary_data.docx]

**Search including key-words**

*Databases: PubMed, Web of Science and Ebscohost (Cinahl, Econlit, PsycINFO)*

PubMed

Filters: Classical Article; Clinical Study; Clinical Trial; Clinical Trial, Phase I; Clinical Trial, Phase II; Clinical Trial, Phase III; Clinical Trial, Phase IV; Comparative Study; Controlled Clinical Trial; Corrected and Republished Article; Dataset; Evaluation Studies; Government Publications; Journal Article; Multicenter Study; Observational Study; Pragmatic Clinical Trial; Randomized Controlled Trial; Research Support, American Recovery and Reinvestment Act; Research Support, N.I.H., Extramural; Research Support, N.I.H., Intramural; Research Support, Non-U.S. Gov't; Research Support, U.S. Gov't, Non-P.H.S.; Research Support, U.S. Gov't, P.H.S.; Research Support, U.S. Government; Publication date from 2007/01/01 to 2016/12/31; Humans; English; Swedish

Depression or anxiety: 158471hits

1. depression [MeSH Terms] OR depressive disorder [MeSH Terms] OR anxiety [MeSH Terms] OR anxiety disorders [MeSH Terms]
2. depress*[TIAB] OR anxiety [TIAB]
3. #1 OR #2

Education: 184346 hits

1. educational achievement [MeSH Terms]
2. educat*[TIAB] OR *educated [TIAB] OR *education [TIAB]
3. #4 OR #5

Employment outcomes: 187009 hits

1. employment [MeSH Terms] OR retirement [MeSH Terms] OR income [MeSH Terms] OR absenteeism [MeSH Terms] OR sick leave [MeSH Terms]
2. employ* [TIAB] OR unemploy* [TIAB] OR retirement [TIAB] OR *income [TIAB] OR income* [TIAB] OR absenteeism [TIAB] OR “sick leave” [TIAB] OR "low salary" [TIAB] OR “high salary” [TIAB] OR demotion [TIAB] OR productivit* [TIAB]
3. ((work OR occupational OR vocational OR employment) [TIAB] AND (status OR outcome OR functioning OR performance) [TIAB])
4. #7 OR #8 OR #9

Study design: 1458263 hits

1. observational [all fields] OR register [all fields] OR retrospective [all fields] OR prospective [all fields] OR follow-up [all fields] OR longterm [all fields] OR long-term [all fields] OR case-control [all fields] OR cohort [all fields] OR cross-sectional [all fields] OR ”population study” [all fields] OR ”population studies” [all fields] OR longitudinal [all fields] OR “register study” [all fields] OR “register studies” [all fields] OR “epidemiologic study” [all fields]

Europe: 300696 hits

1. Europe [MeSH Terms]

Total hits PUBMED: 2045 hits

1. (#3) AND (#6 OR #10) AND (#11) AND (#12)

Web of Science

Indexes=SCI-EXPANDED, SSCI, A&HCI, ESCI Timespan=2007-2016

Depression or anxiety: 90617 hits

1. depress* [title] OR anxiety [title]
2. (animal* [topic] OR mice [topic] OR rats [topic] OR monkey [topic] OR dogs [topic] OR "guinea pigs" [topic])
3. (#14) NOT (#15)

Education: 410537 hits

1. *educat*[topic]

Employment outcomes: 2108957 hits

1. *employ* [topic] OR work* [topic] OR retirement [topic] OR *income* [topic] OR absenteeism [topic] OR “sick leave” [topic] OR "low salary" [topic] OR “high salary” [topic] OR demotion [topic] OR productivit* [topic]
2. ((work OR occupational OR vocational OR employment) [topic] AND (status OR outcome OR functioning OR performance) [topic])
3. #18 OR #19

Study design 1821759 hits

1. observational [topic] OR register [topic] OR retrospective [topic] OR prospective [topic] OR follow-up [topic] OR longterm [topic] OR long-term [topic] OR case-control [topic] OR cohort [topic] OR cross-sectional [topic] OR ”population study” [topic] OR ”population studies” [topic] OR longitudinal [topic] OR “register study” [topic] OR “register studies” [topic] OR “epidemiologic study” [topic]

Total hits WEB OF SCIENCE: 1646 hits

Refined by: DOCUMENT TYPES: ( ARTICLE ) AND COUNTRIES/TERRITORIES: ( ENGLAND OR GREECE OR NETHERLANDS OR PORTUGAL OR GERMANY OR HUNGARY OR SWEDEN OR SLOVAKIA OR SPAIN OR CZECH REPUBLIC OR CYPRUS OR FRANCE OR CROATIA OR ITALY OR ESTONIA OR BULGARIA OR FINLAND OR ROMANIA OR NORWAY OR SLOVENIA OR DENMARK OR SWITZERLAND OR ICELAND OR SCOTLAND OR NORTH IRELAND OR BELGIUM OR AUSTRIA OR POLAND OR WALES OR IRELAND OR LITHUANIA ) AND LANGUAGES: (ENGLISH)^[[1]](#footnote-1)^

1. (#16) AND (#17 OR #20) AND (#21)

Ebscohost (Cinahl, Econlit, PsycINFO)

Limiters - Published Date: 20070101-20161231; Peer Reviewed; Human; Population Group: Human

Depression or anxiety: 168779 hits

1. depress* [ab] OR anxiety [ab]

Education: 207907 hits

1. *educat* [ab]

Employment outcomes: 398578 hits

1. *employ* [ab] OR work* [ab] OR retirement [ab] OR *income* [ab] OR absenteeism [ab] OR “sick leave” [ab] OR "low* salar*" [ab] OR “high* salar*” [ab] OR demotion [ab] OR productivit* [ab]
2. ((work OR occupational OR vocational OR employment) [ab] AND (status OR outcome OR functioning OR performance) [ab])
3. #25 OR #26

Study design: 754000 hits

1. observational [tx] OR register [tx] OR retrospective [tx] OR prospective [tx] OR follow-up [tx] OR longterm [tx] OR long-term [tx] OR case-control [tx] OR cohort [tx] OR cross-sectional [tx] OR ”population study” [tx] OR ”population studies” [tx] OR longitudinal [tx] OR “register study” [tx] OR “register studies” [tx] OR “epidemiologic study” [tx]

Total hits EBSCOHOST: 1535 hits

Narrow by SubjectGeographic: - europe

Narrow by Language: - English (there were no Swedish studies)

1. (#23) AND (#24 OR #27) AND (#28)

*Grey area literature*

The World Bank website

We searched in “Documents and Reports” for publications on topic “Mental health”.

Total hits: 24

The World Health Organization (WHO)

We searched the digital library Iris for publications related to mental health

Subject “depression”

Total hits =17

Subject “anxiety”

Total hits: 9

The UK Department for International Development (DFID)

We searched for publications that contained “depression or anxiety” (published after 20070101)

Total hits: 138

The Organization for Economic Co-operation and Development (OECD)

We searched the OECD iLibrary in the quick search browser for “depression or anxiety” limited to publications between 2007-2016

Total hits: 5

1. No Swedish studies [↑](#footnote-ref-1)
